# Phase I study of alphaviral vector (AVX701) in colorectal cancer patients: comparison of immune responses in stage III and stage IV patients

**DOI:** 10.1186/2051-1426-3-S2-P444

**Published:** 2015-11-04

**Authors:** Michael A Morse, Amy Hobeika, William Gwin, Takuya Osada, Jonathan Gelles, Christel Rushing, Donna Niedzwiecki, H Kim Lyerly

**Affiliations:** 1 Duke University Medical Center, Durham, NC, USA; 2 Alphavax, Durham, NC, USA

## Background

AVX701 is an alphaviral replicon particle vaccine expressing a modified carcinoembryonic antigen gene (CEA(6D)). We previously reported the preliminary results of a Phase I/II study of AVX701 in metastatic colorectal cancer patients (Morse, J Clin Invest. 2010; 120: 3234–3241). Herein, we now report long-term survival for these patients and compare their T cell and antibody responses with the immune responses in a new cohort of stage III colorectal cancer patients immunized similarly with AVX701.

## Methods

Twenty-eight (28) heavily pretreated patients with predominantly stage IV colorectal cancer were enrolled to 3 dose levels of AVX701 (highest dose 4 × 10^8^ IU i.m. every 3 weeks for 4 immunizations). Subsequently 6 patients with stage III colorectal cancer who had completed adjuvant chemotherapy were enrolled to the same regimen. Peripheral blood from before and after all immunizations was analyzed for immune responses (IR): anti-CEA antibody by ELISA and CEA-specific T cells by ELISPOT.

## Results

For the entire metastatic cohort (n=28; 23 deaths), median OS was 16 months, 95% CI (8, 25). In those treated at the highest dose (n=19; 14 deaths), there was a trend for a longer median OS among CEA-specific T cell (ELISPOT) immune responders vs nonresponders (not reached vs 13 mo, Log rank p=0.10). ELISA data were too sparse to study OS in the metastatic patients. The difference between maximum anti-CEA antibody (reciprocal) titer and baseline was significantly higher in the Stage III cohort (Normal Scores Test, 2-sided p=0.01) as was the proportion of patients exhibiting IR by ELISA (100% vs 45.1%; Chi-square p=0.02). In the stage III cohort, mean ELISPOT values were significantly higher (F-test p = 0.003) and the rate of CEA-specific T cell response by ELISPOT tended to be higher (67% and 30%; Exact Pearson Chi-square, p=0.16) than in the stage IV cohort.

## Conclusions

T cell responses following immunization with AVX701 were associated with a trend for longer survival in patients with predominantly stage IV colorectal cancer. The magnitude of antibody response and rate of T cell response tended to be greater in the stage III than stage IV patients which may reflect a less immunosuppressive milieu in patients without measurable disease. We hypothesize that future combinations with checkpoint blockade may enhance efficacy in more immunosuppressive environments.

